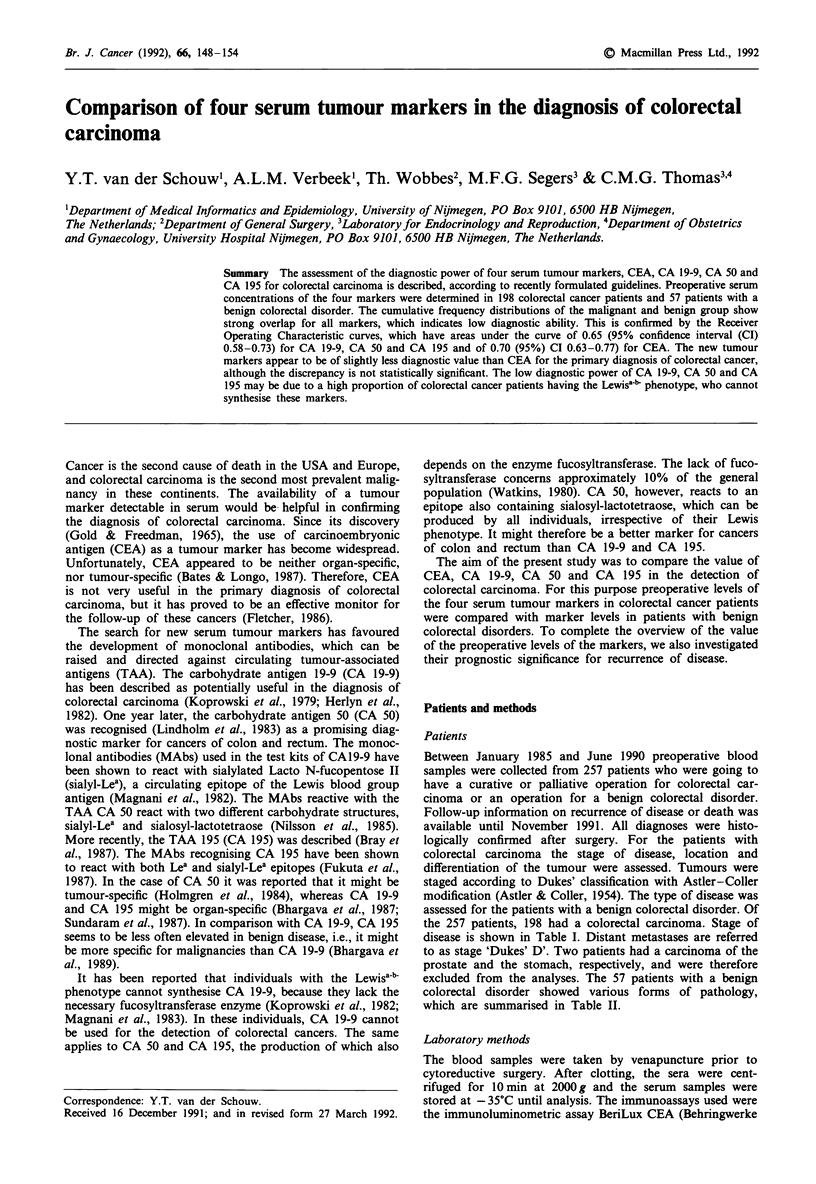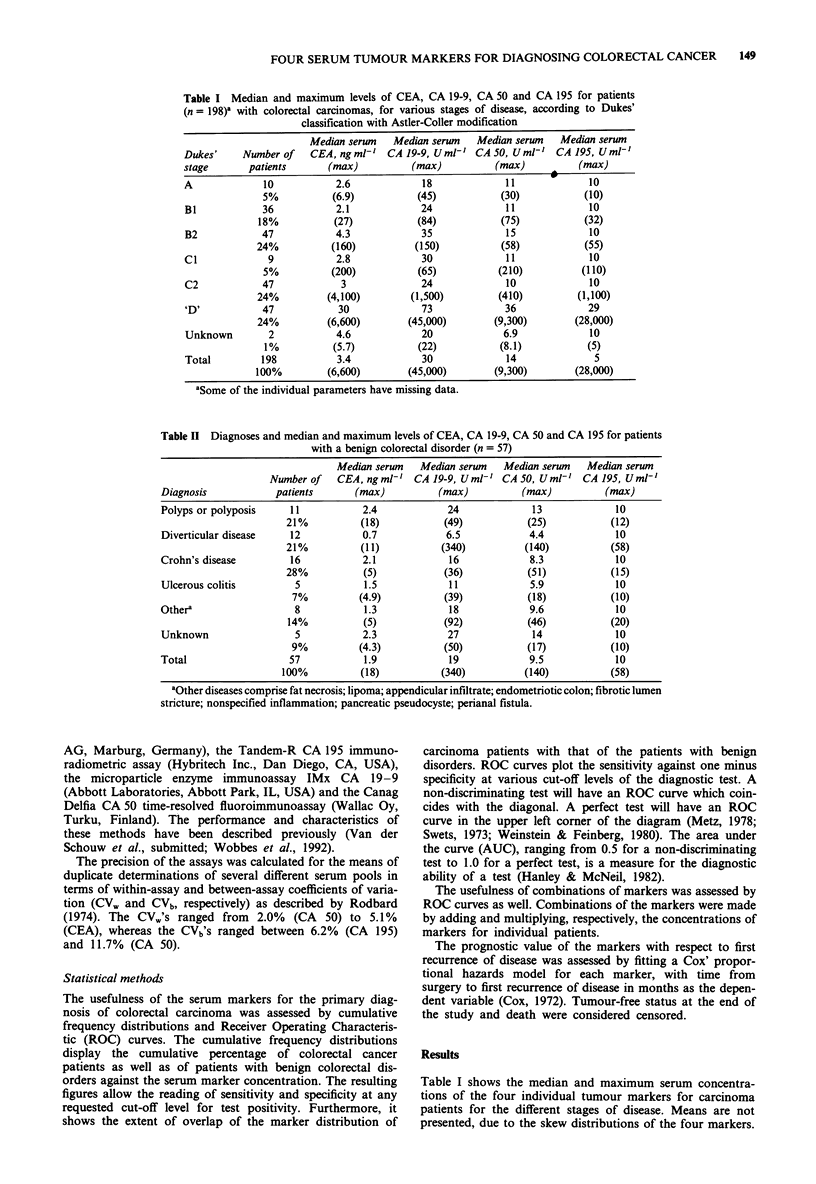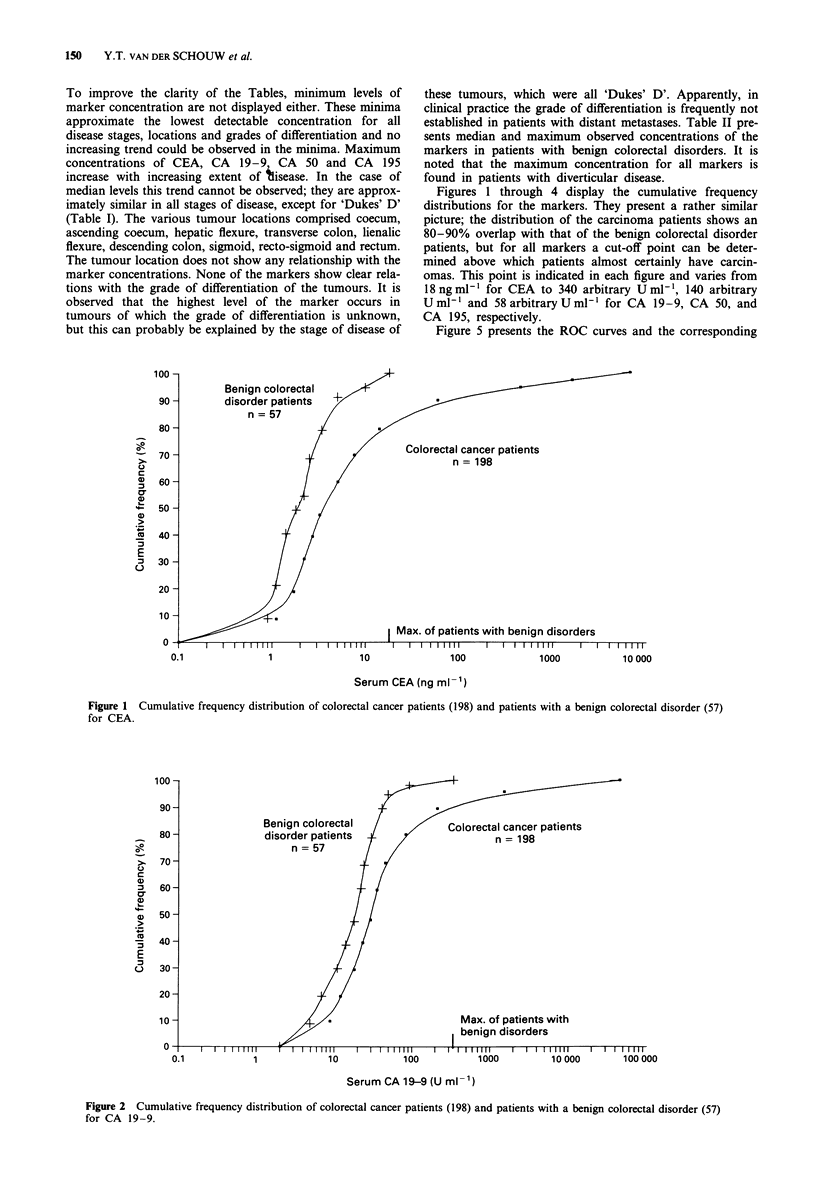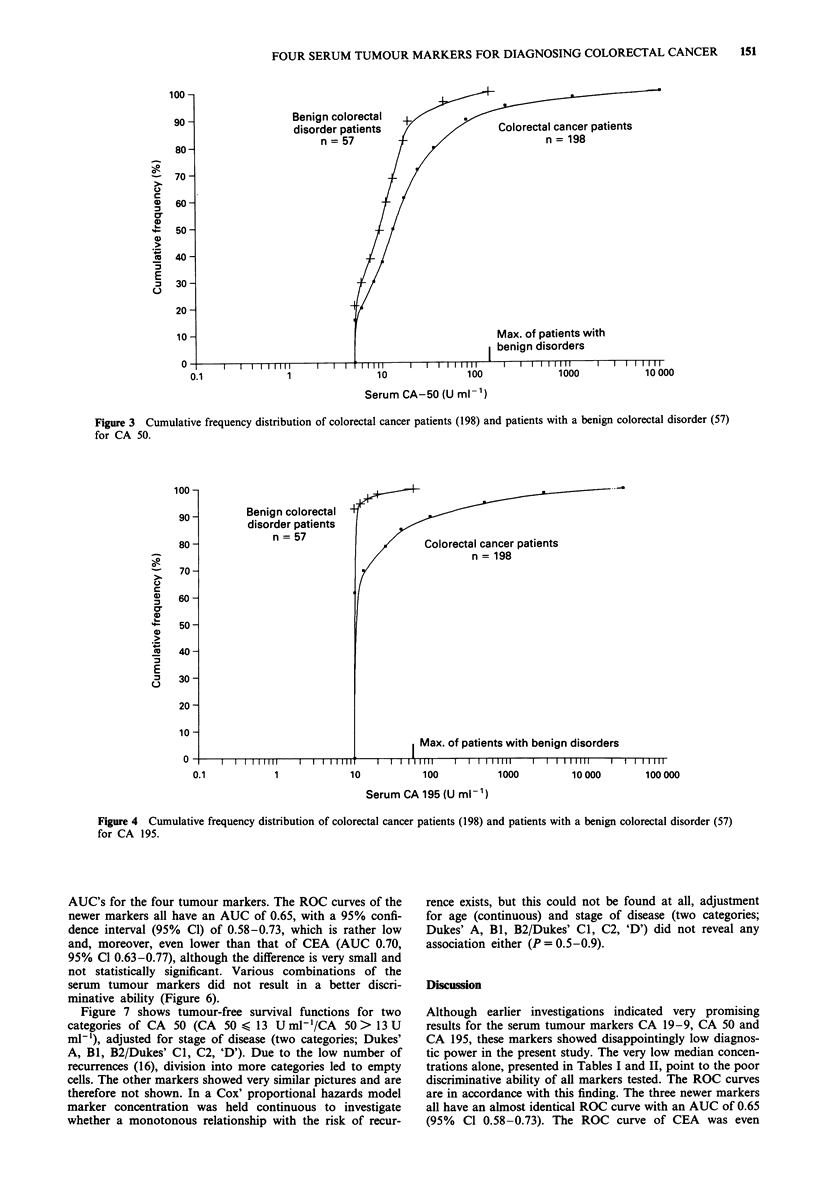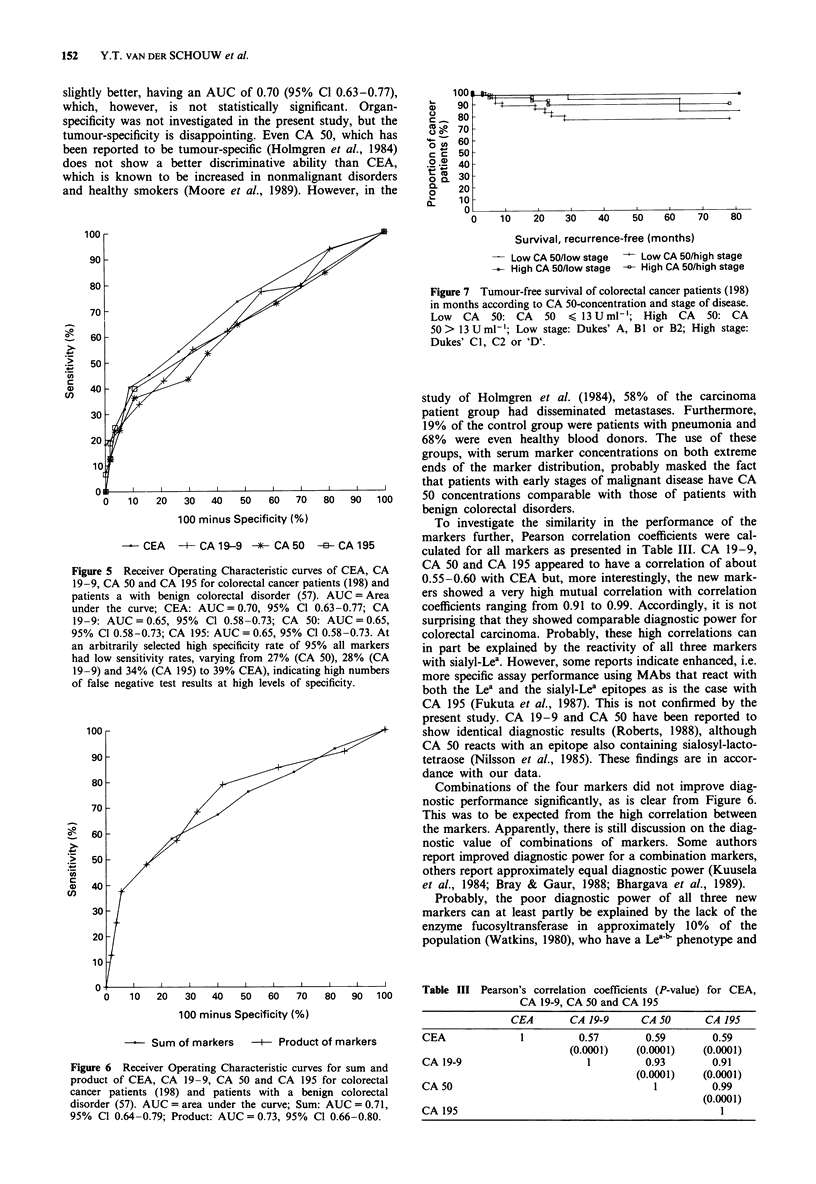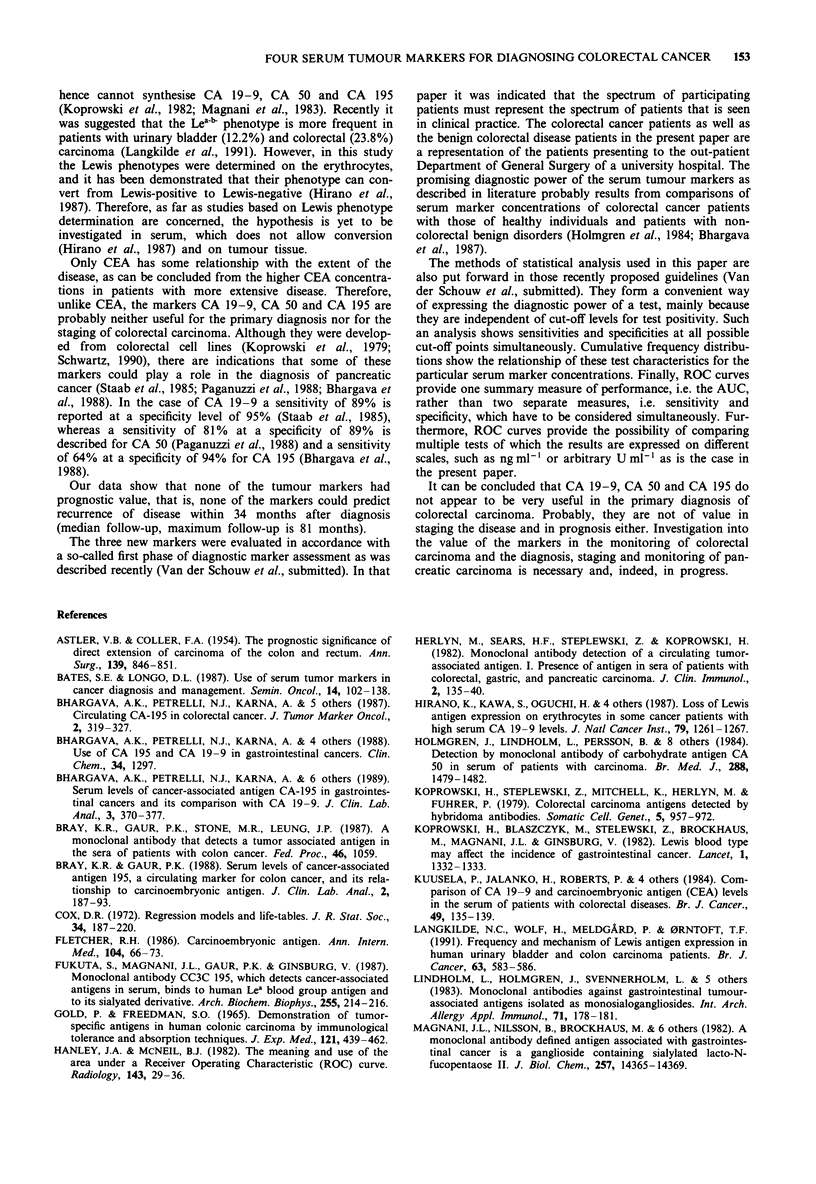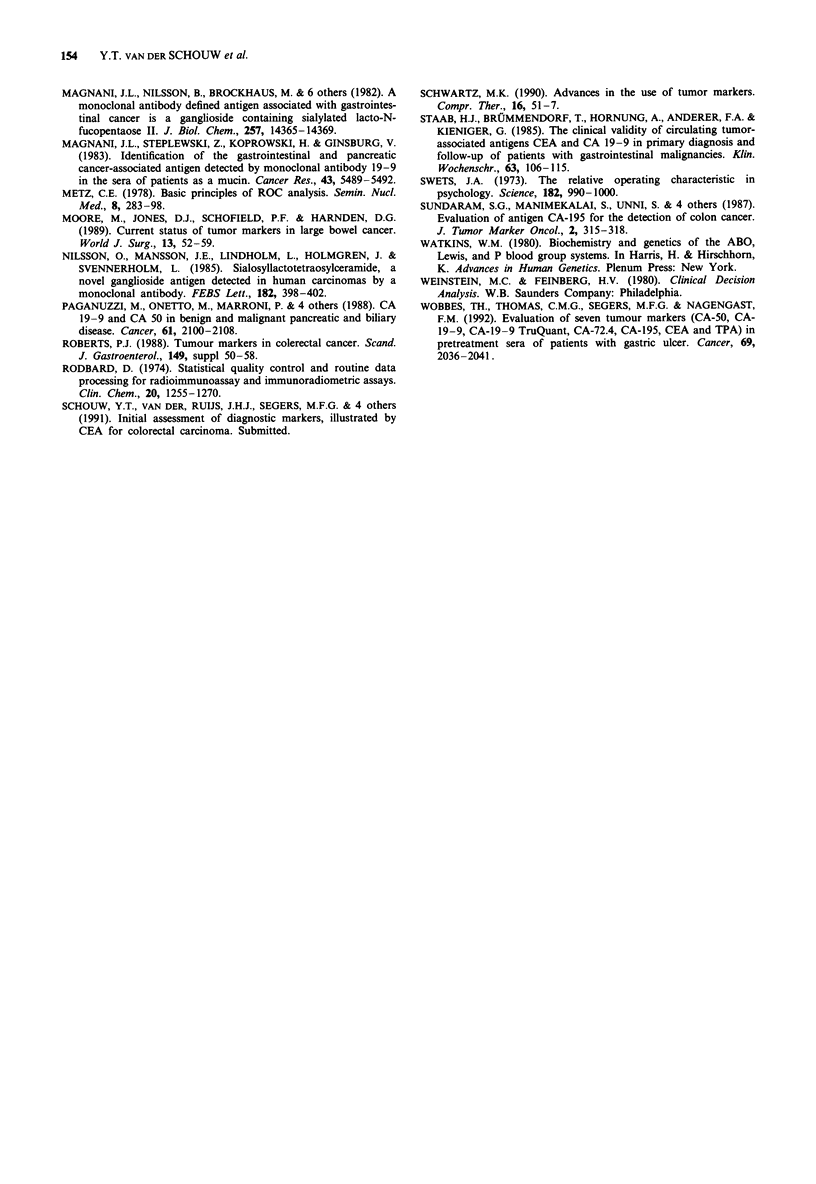# Comparison of four serum tumour markers in the diagnosis of colorectal carcinoma.

**DOI:** 10.1038/bjc.1992.233

**Published:** 1992-07

**Authors:** Y. T. van der Schouw, A. L. Verbeek, T. Wobbes, M. F. Segers, C. M. Thomas

**Affiliations:** Department of Medical Informatics and Epidemiology, University of Nijmegen, The Netherlands.

## Abstract

The assessment of the diagnostic power of four serum tumour markers, CEA, CA 19-9, CA 50 and CA 195 for colorectal carcinoma is described, according to recently formulated guidelines. Preoperative serum concentrations of the four markers were determined in 198 colorectal cancer patients and 57 patients with a benign colorectal disorder. The cumulative frequency distributions of the malignant and benign group show strong overlap for all markers, which indicates low diagnostic ability. This is confirmed by the Receiver Operating Characteristic curves, which have areas under the curve of 0.65 (95% confidence interval (CI) 0.58-0.73) for CA 19-9, CA 50 and CA 195 and of 0.70 (95%) CI 0.63-0.77) for CEA. The new tumour markers appear to be of slightly less diagnostic value than CEA for the primary diagnosis of colorectal cancer, although the discrepancy is not statistically significant. The low diagnostic power of CA 19-9, CA 50 and CA 195 may be due to a high proportion of colorectal cancer patients having the Lewis(a-b-) phenotype, who cannot synthesise these markers.


Br. J. Cancer (1992), 66, 148–154 © Macmillan Press Ltd., 1992

### Comparison of four serum tumour markers in the diagnosis of colorectal carcinoma

Y.T. van der Schouw[1], A.L.M. Verbeek[1], Th. Wobbes[2], M.F.G. Segers[3] & C.M.G. Thomas[3,4]

[1]*Department of Medical Informatics and Epidemiology, University of Nijmegen, PO Box 9101, 6500 HB Nijmegen, The Netherlands;* [2]*Department of General Surgery,* [3]*Laboratory for Endocrinology and Reproduction,* [4]*Department of Obstetrics and Gynaecology, University Hospital Nijmegen, PO Box 9101, 6500 HB Nijmegen, The Netherlands.*

**Summary** The assessment of the diagnostic power of four serum tumour markers, CEA, CA 19-9, CA 50 and CA 195 for colorectal carcinoma is described, according to recently formulated guidelines. Preoperative serum concentrations of the four markers were determined in 198 colorectal cancer patients and 57 patients with a benign colorectal disorder. The cumulative frequency distributions of the malignant and benign group show strong overlap for all markers, which indicates low diagnostic ability. This is confirmed by the Receiver Operating Characteristic curves, which have areas under the curve of 0.65 (95% confidence interval (CI) 0.58–0.73) for CA 19-9, CA 50 and CA 195 and of 0.70 (95%) CI 0.63–0.77) for CEA. The new tumour markers appear to be of slightly less diagnostic value than CEA for the primary diagnosis of colorectal cancer, although the discrepancy is not statistically significant. The low diagnostic power of CA 19-9, CA 50 and CA 195 may be due to a high proportion of colorectal cancer patients having the Lewis$^{a-b-}$ phenotype, who cannot synthesise these markers.

Cancer is the second cause of death in the USA and Europe, and colorectal carcinoma is the second most prevalent malignancy in these continents. The availability of a tumour marker detectable in serum would be helpful in confirming the diagnosis of colorectal carcinoma. Since its discovery (Gold & Freedman, 1965), the use of carcinoembryonic antigen (CEA) as a tumour marker has become widespread. Unfortunately, CEA appeared to be neither organ-specific, nor tumour-specific (Bates & Longo, 1987). Therefore, CEA is not very useful in the primary diagnosis of colorectal carcinoma, but it has proved to be an effective monitor for the follow-up of these cancers (Fletcher, 1986).

The search for new serum tumour markers has favoured the development of monoclonal antibodies, which can be raised and directed against circulating tumour-associated antigens (TAA). The carbohydrate antigen 19-9 (CA 19-9) has been described as potentially useful in the diagnosis of colorectal carcinoma (Koprowski *et al.*, 1979; Herlyn *et al.*, 1982). One year later, the carbohydrate antigen 50 (CA 50) was recognised (Lindholm *et al.*, 1983) as a promising diagnostic marker for cancers of colon and rectum. The monoclonal antibodies (MAbs) used in the test kits of CA19-9 have been shown to react with sialylated Lacto N-fucopentose II (sialyl-Le$^a$), a circulating epitope of the Lewis blood group antigen (Magnani *et al.*, 1982). The MAbs reactive with the TAA CA 50 react with two different carbohydrate structures, sialyl-Le$^a$ and sialosyl-lactotetraose (Nilsson *et al.*, 1985). More recently, the TAA 195 (CA 195) was described (Bray *et al.*, 1987). The MAbs recognising CA 195 have been shown to react with both Le$^a$ and sialyl-Le$^a$ epitopes (Fukuta *et al.*, 1987). In the case of CA 50 it was reported that it might be tumour-specific (Holmgren *et al.*, 1984), whereas CA 19-9 and CA 195 might be organ-specific (Bhargava *et al.*, 1987; Sundaram *et al.*, 1987). In comparison with CA 19-9, CA 195 seems to be less often elevated in benign disease, i.e., it might be more specific for malignancies than CA 19-9 (Bhargava *et al.*, 1989).

It has been reported that individuals with the Lewis$^{a-b-}$ phenotype cannot synthesise CA 19-9, because they lack the necessary fucosyltransferase enzyme (Koprowski *et al.*, 1982; Magnani *et al.*, 1983). In these individuals, CA 19-9 cannot be used for the detection of colorectal cancers. The same applies to CA 50 and CA 195, the production of which also depends on the enzyme fucosyltransferase. The lack of fucosyltransferase concerns approximately 10% of the general population (Watkins, 1980). CA 50, however, reacts to an epitope also containing sialosyl-lactotetraose, which can be produced by all individuals, irrespective of their Lewis phenotype. It might therefore be a better marker for cancers of colon and rectum than CA 19-9 and CA 195.

The aim of the present study was to compare the value of CEA, CA 19-9, CA 50 and CA 195 in the detection of colorectal carcinoma. For this purpose preoperative levels of the four serum tumour markers in colorectal cancer patients were compared with marker levels in patients with benign colorectal disorders. To complete the overview of the value of the preoperative levels of the markers, we also investigated their prognostic significance for recurrence of disease.

### Patients and methods

### *Patients*

Between January 1985 and June 1990 preoperative blood samples were collected from 257 patients who were going to have a curative or palliative operation for colorectal carcinoma or an operation for a benign colorectal disorder. Follow-up information on recurrence of disease or death was available until November 1991. All diagnoses were histologically confirmed after surgery. For the patients with colorectal carcinoma the stage of disease, location and differentiation of the tumour were assessed. Tumours were staged according to Dukes' classification with Astler–Coller modification (Astler & Coller, 1954). The type of disease was assessed for the patients with a benign colorectal disorder. Of the 257 patients, 198 had a colorectal carcinoma. Stage of disease is shown in Table I. Distant metastases are referred to as stage 'Dukes' D'. Two patients had a carcinoma of the prostate and the stomach, respectively, and were therefore excluded from the analyses. The 57 patients with a benign colorectal disorder showed various forms of pathology, which are summarised in Table II.

### *Laboratory methods*

The blood samples were taken by venapuncture prior to cytoreductive surgery. After clotting, the sera were centrifuged for 10 min at 2000 *g* and the serum samples were stored at −35°C until analysis. The immunoassays used were the immunoluminometric assay BeriLux CEA (Behringwerke

Correspondence: Y.T. van der Schouw.
Received 16 December 1991; and in revised form 27 March 1992.

**Table I** Median and maximum levels of CEA, CA 19-9, CA 50 and CA 195 for patients ($n = 198$)[a] with colorectal carcinomas, for various stages of disease, according to Dukes' classification with Astler-Coller modification

| *Dukes' stage* | *Number of patients* | *Median serum CEA, ng $ml^{-1}$ (max)* | *Median serum CA 19-9, U $ml^{-1}$ (max)* | *Median serum CA 50, U $ml^{-1}$ (max)* | *Median serum CA 195, U $ml^{-1}$ (max)* |
|---|---|---|---|---|---|
| A | 10 | 2.6 | 18 | 11 | 10 |
| | 5% | (6.9) | (45) | (30) | (10) |
| B1 | 36 | 2.1 | 24 | 11 | 10 |
| | 18% | (27) | (84) | (75) | (32) |
| B2 | 47 | 4.3 | 35 | 15 | 10 |
| | 24% | (160) | (150) | (58) | (55) |
| C1 | 9 | 2.8 | 30 | 11 | 10 |
| | 5% | (200) | (65) | (210) | (110) |
| C2 | 47 | 3 | 24 | 10 | 10 |
| | 24% | (4,100) | (1,500) | (410) | (1,100) |
| 'D' | 47 | 30 | 73 | 36 | 29 |
| | 24% | (6,600) | (45,000) | (9,300) | (28,000) |
| Unknown | 2 | 4.6 | 20 | 6.9 | 10 |
| | 1% | (5.7) | (22) | (8.1) | (5) |
| Total | 198 | 3.4 | 30 | 14 | 5 |
| | 100% | (6,600) | (45,000) | (9,300) | (28,000) |

[a]Some of the individual parameters have missing data.

**Table II** Diagnoses and median and maximum levels of CEA, CA 19-9, CA 50 and CA 195 for patients with a benign colorectal disorder ($n = 57$)

| *Diagnosis* | *Number of patients* | *Median serum CEA, ng $ml^{-1}$ (max)* | *Median serum CA 19-9, U $ml^{-1}$ (max)* | *Median serum CA 50, U $ml^{-1}$ (max)* | *Median serum CA 195, U $ml^{-1}$ (max)* |
|---|---|---|---|---|---|
| Polyps or polyposis | 11 | 2.4 | 24 | 13 | 10 |
| | 21% | (18) | (49) | (25) | (12) |
| Diverticular disease | 12 | 0.7 | 6.5 | 4.4 | 10 |
| | 21% | (11) | (340) | (140) | (58) |
| Crohn's disease | 16 | 2.1 | 16 | 8.3 | 10 |
| | 28% | (5) | (36) | (51) | (15) |
| Ulcerous colitis | 5 | 1.5 | 11 | 5.9 | 10 |
| | 7% | (4.9) | (39) | (18) | (10) |
| Other[a] | 8 | 1.3 | 18 | 9.6 | 10 |
| | 14% | (5) | (92) | (46) | (20) |
| Unknown | 5 | 2.3 | 27 | 14 | 10 |
| | 9% | (4.3) | (50) | (17) | (10) |
| Total | 57 | 1.9 | 19 | 9.5 | 10 |
| | 100% | (18) | (340) | (140) | (58) |

[a]Other diseases comprise fat necrosis; lipoma; appendicular infiltrate; endometriotic colon; fibrotic lumen stricture; nonspecified inflammation; pancreatic pseudocyste; perianal fistula.

AG, Marburg, Germany), the Tandem-R CA 195 immunoradiometric assay (Hybritech Inc., Dan Diego, CA, USA), the microparticle enzyme immunoassay IMx CA 19–9 (Abbott Laboratories, Abbott Park, IL, USA) and the Canag Delfia CA 50 time-resolved fluoroimmunoassay (Wallac Oy, Turku, Finland). The performance and characteristics of these methods have been described previously (Van der Schouw *et al.*, submitted; Wobbes *et al.*, 1992).

The precision of the assays was calculated for the means of duplicate determinations of several different serum pools in terms of within-assay and between-assay coefficients of variation ($CV_w$ and $CV_b$, respectively) as described by Rodbard (1974). The $CV_w$'s ranged from 2.0% (CA 50) to 5.1% (CEA), whereas the $CV_b$'s ranged between 6.2% (CA 195) and 11.7% (CA 50).

### *Statistical methods*

The usefulness of the serum markers for the primary diagnosis of colorectal carcinoma was assessed by cumulative frequency distributions and Receiver Operating Characteristic (ROC) curves. The cumulative frequency distributions display the cumulative percentage of colorectal cancer patients as well as of patients with benign colorectal disorders against the serum marker concentration. The resulting figures allow the reading of sensitivity and specificity at any requested cut-off level for test positivity. Furthermore, it shows the extent of overlap of the marker distribution of carcinoma patients with that of the patients with benign disorders. ROC curves plot the sensitivity against one minus specificity at various cut-off levels of the diagnostic test. A non-discriminating test will have an ROC curve which coincides with the diagonal. A perfect test will have an ROC curve in the upper left corner of the diagram (Metz, 1978; Swets, 1973; Weinstein & Feinberg, 1980). The area under the curve (AUC), ranging from 0.5 for a non-discriminating test to 1.0 for a perfect test, is a measure for the diagnostic ability of a test (Hanley & McNeil, 1982).

The usefulness of combinations of markers was assessed by ROC curves as well. Combinations of the markers were made by adding and multiplying, respectively, the concentrations of markers for individual patients.

The prognostic value of the markers with respect to first recurrence of disease was assessed by fitting a Cox' proportional hazards model for each marker, with time from surgery to first recurrence of disease in months as the dependent variable (Cox, 1972). Tumour-free status at the end of the study and death were considered censored.

### Results

Table I shows the median and maximum serum concentrations of the four individual tumour markers for carcinoma patients for the different stages of disease. Means are not presented, due to the skew distributions of the four markers.

To improve the clarity of the Tables, minimum levels of marker concentration are not displayed either. These minima approximate the lowest detectable concentration for all disease stages, locations and grades of differentiation and no increasing trend could be observed in the minima. Maximum concentrations of CEA, CA 19-9, CA 50 and CA 195 increase with increasing extent of disease. In the case of median levels this trend cannot be observed; they are approximately similar in all stages of disease, except for 'Dukes' D' (Table I). The various tumour locations comprised coecum, ascending coecum, hepatic flexure, transverse colon, lienalic flexure, descending colon, sigmoid, recto-sigmoid and rectum. The tumour location does not show any relationship with the marker concentrations. None of the markers show clear relations with the grade of differentiation of the tumours. It is observed that the highest level of the marker occurs in tumours of which the grade of differentiation is unknown, but this can probably be explained by the stage of disease of these tumours, which were all 'Dukes' D'. Apparently, in clinical practice the grade of differentiation is frequently not established in patients with distant metastases. Table II presents median and maximum observed concentrations of the markers in patients with benign colorectal disorders. It is noted that the maximum concentration for all markers is found in patients with diverticular disease.

Figures 1 through 4 display the cumulative frequency distributions for the markers. They present a rather similar picture; the distribution of the carcinoma patients shows an 80-90% overlap with that of the benign colorectal disorder patients, but for all markers a cut-off point can be determined above which patients almost certainly have carcinomas. This point is indicated in each figure and varies from 18 ng $ml^{-1}$ for CEA to 340 arbitrary U $ml^{-1}$, 140 arbitrary U $ml^{-1}$ and 58 arbitrary U $ml^{-1}$ for CA 19-9, CA 50, and CA 195, respectively.

Figure 5 presents the ROC curves and the corresponding

**Figure 1** Cumulative frequency distribution of colorectal cancer patients (198) and patients with a benign colorectal disorder (57) for CEA.

**Figure 2** Cumulative frequency distribution of colorectal cancer patients (198) and patients with a benign colorectal disorder (57) for CA 19-9.

**Figure 3** Cumulative frequency distribution of colorectal cancer patients (198) and patients with a benign colorectal disorder (57) for CA 50.

**Figure 4** Cumulative frequency distribution of colorectal cancer patients (198) and patients with a benign colorectal disorder (57) for CA 195.

AUC's for the four tumour markers. The ROC curves of the newer markers all have an AUC of 0.65, with a 95% confidence interval (95% Cl) of 0.58–0.73, which is rather low and, moreover, even lower than that of CEA (AUC 0.70, 95% Cl 0.63–0.77), although the difference is very small and not statistically significant. Various combinations of the serum tumour markers did not result in a better discriminative ability (Figure 6).

Figure 7 shows tumour-free survival functions for two categories of CA 50 (CA 50 $\leqslant$ 13 U ml$^{-1}$/CA 50 $>$ 13 U ml$^{-1}$), adjusted for stage of disease (two categories; Dukes' A, B1, B2/Dukes' C1, C2, 'D'). Due to the low number of recurrences (16), division into more categories led to empty cells. The other markers showed very similar pictures and are therefore not shown. In a Cox' proportional hazards model marker concentration was held continuous to investigate whether a monotonous relationship with the risk of recurrence exists, but this could not be found at all, adjustment for age (continuous) and stage of disease (two categories; Dukes' A, B1, B2/Dukes' C1, C2, 'D') did not reveal any association either ($P = 0.5–0.9$).

### Discussion

Although earlier investigations indicated very promising results for the serum tumour markers CA 19–9, CA 50 and CA 195, these markers showed disappointingly low diagnostic power in the present study. The very low median concentrations alone, presented in Tables I and II, point to the poor discriminative ability of all markers tested. The ROC curves are in accordance with this finding. The three newer markers all have an almost identical ROC curve with an AUC of 0.65 (95% Cl 0.58–0.73). The ROC curve of CEA was even slightly better, having an AUC of 0.70 (95% Cl 0.63–0.77), which, however, is not statistically significant. Organ-specificity was not investigated in the present study, but the tumour-specificity is disappointing. Even CA 50, which has been reported to be tumour-specific (Holmgren et al., 1984) does not show a better discriminative ability than CEA, which is known to be increased in nonmalignant disorders and healthy smokers (Moore et al., 1989). However, in the study of Holmgren et al. (1984), 58% of the carcinoma patient group had disseminated metastases. Furthermore, 19% of the control group were patients with pneumonia and 68% were even healthy blood donors. The use of these groups, with serum marker concentrations on both extreme ends of the marker distribution, probably masked the fact that patients with early stages of malignant disease have CA 50 concentrations comparable with those of patients with benign colorectal disorders.

**Figure 5** Receiver Operating Characteristic curves of CEA, CA 19-9, CA 50 and CA 195 for colorectal cancer patients (198) and patients a with benign colorectal disorder (57). AUC = Area under the curve; CEA: AUC = 0.70, 95% Cl 0.63–0.77; CA 19-9: AUC = 0.65, 95% Cl 0.58–0.73; CA 50: AUC = 0.65, 95% Cl 0.58–0.73; CA 195: AUC = 0.65, 95% Cl 0.58–0.73. At an arbitrarily selected high specificity rate of 95% all markers had low sensitivity rates, varying from 27% (CA 50), 28% (CA 19-9) and 34% (CA 195) to 39% CEA), indicating high numbers of false negative test results at high levels of specificity.

**Figure 6** Receiver Operating Characteristic curves for sum and product of CEA, CA 19-9, CA 50 and CA 195 for colorectal cancer patients (198) and patients with a benign colorectal disorder (57). AUC = area under the curve; Sum: AUC = 0.71, 95% Cl 0.64–0.79; Product: AUC = 0.73, 95% Cl 0.66–0.80.

**Figure 7** Tumour-free survival of colorectal cancer patients (198) in months according to CA 50-concentration and stage of disease. Low CA 50: CA 50 $\leq 13\,U\,ml^{-1}$; High CA 50: CA 50 $> 13\,U\,ml^{-1}$; Low stage: Dukes' A, B1 or B2; High stage: Dukes' C1, C2 or 'D'.

To investigate the similarity in the performance of the markers further, Pearson correlation coefficients were calculated for all markers as presented in Table III. CA 19-9, CA 50 and CA 195 appeared to have a correlation of about 0.55–0.60 with CEA but, more interestingly, the new markers showed a very high mutual correlation with correlation coefficients ranging from 0.91 to 0.99. Accordingly, it is not surprising that they showed comparable diagnostic power for colorectal carcinoma. Probably, these high correlations can in part be explained by the reactivity of all three markers with sialyl-$Le^a$. However, some reports indicate enhanced, i.e. more specific assay performance using MAbs that react with both the $Le^a$ and the sialyl-$Le^a$ epitopes as is the case with CA 195 (Fukuta et al., 1987). This is not confirmed by the present study. CA 19-9 and CA 50 have been reported to show identical diagnostic results (Roberts, 1988), although CA 50 reacts with an epitope also containing sialosyl-lactotetraose (Nilsson et al., 1985). These findings are in accordance with our data.

Combinations of the four markers did not improve diagnostic performance significantly, as is clear from Figure 6. This was to be expected from the high correlation between the markers. Apparently, there is still discussion on the diagnostic value of combinations of markers. Some authors report improved diagnostic power for a combination markers, others report approximately equal diagnostic power (Kuusela et al., 1984; Bray & Gaur, 1988; Bhargava et al., 1989).

Probably, the poor diagnostic power of all three new markers can at least partly be explained by the lack of the enzyme fucosyltransferase in approximately 10% of the population (Watkins, 1980), who have a $Le^{a-b-}$ phenotype and

**Table III** Pearson's correlation coefficients (P-value) for CEA, CA 19-9, CA 50 and CA 195

| | CEA | CA 19-9 | CA 50 | CA 195 |
|---|---|---|---|---|
| CEA | 1 | 0.57 (0.0001) | 0.59 (0.0001) | 0.59 (0.0001) |
| CA 19-9 | | 1 | 0.93 (0.0001) | 0.91 (0.0001) |
| CA 50 | | | 1 | 0.99 (0.0001) |
| CA 195 | | | | 1 |

hence cannot synthesise CA 19-9, CA 50 and CA 195 (Koprowski *et al.*, 1982; Magnani *et al.*, 1983). Recently it was suggested that the $Le^{a-b-}$ phenotype is more frequent in patients with urinary bladder (12.2%) and colorectal (23.8%) carcinoma (Langkilde *et al.*, 1991). However, in this study the Lewis phenotypes were determined on the erythrocytes, and it has been demonstrated that their phenotype can convert from Lewis-positive to Lewis-negative (Hirano *et al.*, 1987). Therefore, as far as studies based on Lewis phenotype determination are concerned, the hypothesis is yet to be investigated in serum, which does not allow conversion (Hirano *et al.*, 1987) and on tumour tissue.

Only CEA has some relationship with the extent of the disease, as can be concluded from the higher CEA concentrations in patients with more extensive disease. Therefore, unlike CEA, the markers CA 19-9, CA 50 and CA 195 are probably neither useful for the primary diagnosis nor for the staging of colorectal carcinoma. Although they were developed from colorectal cell lines (Koprowski *et al.*, 1979; Schwartz, 1990), there are indications that some of these markers could play a role in the diagnosis of pancreatic cancer (Staab *et al.*, 1985; Paganuzzi *et al.*, 1988; Bhargava *et al.*, 1988). In the case of CA 19-9 a sensitivity of 89% is reported at a specificity level of 95% (Staab *et al.*, 1985), whereas a sensitivity of 81% at a specificity of 89% is described for CA 50 (Paganuzzi *et al.*, 1988) and a sensitivity of 64% at a specificity of 94% for CA 195 (Bhargava *et al.*, 1988).

Our data show that none of the tumour markers had prognostic value, that is, none of the markers could predict recurrence of disease within 34 months after diagnosis (median follow-up, maximum follow-up is 81 months).

The three new markers were evaluated in accordance with a so-called first phase of diagnostic marker assessment as was described recently (Van der Schouw *et al.*, submitted). In that paper it was indicated that the spectrum of participating patients must represent the spectrum of patients that is seen in clinical practice. The colorectal cancer patients as well as the benign colorectal disease patients in the present paper are a representation of the patients presenting to the out-patient Department of General Surgery of a university hospital. The promising diagnostic power of the serum tumour markers as described in literature probably results from comparisons of serum marker concentrations of colorectal cancer patients with those of healthy individuals and patients with non-colorectal benign disorders (Holmgren *et al.*, 1984; Bhargava *et al.*, 1987).

The methods of statistical analysis used in this paper are also put forward in those recently proposed guidelines (Van der Schouw *et al.*, submitted). They form a convenient way of expressing the diagnostic power of a test, mainly because they are independent of cut-off levels for test positivity. Such an analysis shows sensitivities and specificities at all possible cut-off points simultaneously. Cumulative frequency distributions show the relationship of these test characteristics for the particular serum marker concentrations. Finally, ROC curves provide one summary measure of performance, i.e. the AUC, rather than two separate measures, i.e. sensitivity and specificity, which have to be considered simultaneously. Furthermore, ROC curves provide the possibility of comparing multiple tests of which the results are expressed on different scales, such as $ng\ ml^{-1}$ or arbitrary $U\ ml^{-1}$ as is the case in the present paper.

It can be concluded that CA 19-9, CA 50 and CA 195 do not appear to be very useful in the primary diagnosis of colorectal carcinoma. Probably, they are not of value in staging the disease and in prognosis either. Investigation into the value of the markers in the monitoring of colorectal carcinoma and the diagnosis, staging and monitoring of pancreatic carcinoma is necessary and, indeed, in progress.

### References

ASTLER, V.B. & COLLER, F.A. (1954). The prognostic significance of direct extension of carcinoma of the colon and rectum. *Ann. Surg.*, **139**, 846-851.

BATES, S.E. & LONGO, D.L. (1987). Use of serum tumor markers in cancer diagnosis and management. *Semin. Oncol.*, **14**, 102-138.

BHARGAVA, A.K., PETRELLI, N.J., KARNA, A. & 5 others (1987). Circulating CA-195 in colorectal cancer. *J. Tumor Marker Oncol.*, **2**, 319-327.

BHARGAVA, A.K., PETRELLI, N.J., KARNA, A. & 4 others (1988). Use of CA 195 and CA 19-9 in gastrointestinal cancers. *Clin. Chem.*, **34**, 1297.

BHARGAVA, A.K., PETRELLI, N.J., KARNA, A. & 6 others (1989). Serum levels of cancer-associated antigen CA-195 in gastrointestinal cancers and its comparison with CA 19-9. *J. Clin. Lab. Anal.*, **3**, 370-377.

BRAY, K.R., GAUR, P.K., STONE, M.R., LEUNG, J.P. (1987). A monoclonal antibody that detects a tumor associated antigen in the sera of patients with colon cancer. *Fed. Proc.*, **46**, 1059.

BRAY, K.R. & GAUR, P.K. (1988). Serum levels of cancer-associated antigen 195, a circulating marker for colon cancer, and its relationship to carcinoembryonic antigen. *J. Clin. Lab. Anal.*, **2**, 187-93.

COX, D.R. (1972). Regression models and life-tables. *J. R. Stat. Soc.*, **34**, 187-220.

FLETCHER, R.H. (1986). Carcinoembryonic antigen. *Ann. Intern. Med.*, **104**, 66-73.

FUKUTA, S., MAGNANI, J.L., GAUR, P.K. & GINSBURG, V. (1987). Monoclonal antibody CC3C 195, which detects cancer-associated antigens in serum, binds to human $Le^a$ blood group antigen and to its sialyated derivative. *Arch. Biochem. Biophys.*, **255**, 214-216.

GOLD, P. & FREEDMAN, S.O. (1965). Demonstration of tumor-specific antigens in human colonic carcinoma by immunological tolerance and absorption techniques. *J. Exp. Med.*, **121**, 439-462.

HANLEY, J.A. & MCNEIL, B.J. (1982). The meaning and use of the area under a Receiver Operating Characteristic (ROC) curve. *Radiology*, **143**, 29-36.

HERLYN, M., SEARS, H.F., STEPLEWSKI, Z. & KOPROWSKI, H. (1982). Monoclonal antibody detection of a circulating tumor-associated antigen. I. Presence of antigen in sera of patients with colorectal, gastric, and pancreatic carcinoma. *J. Clin. Immunol.*, **2**, 135-40.

HIRANO, K., KAWA, S., OGUCHI, H. & 4 others (1987). Loss of Lewis antigen expression on erythrocytes in some cancer patients with high serum CA 19-9 levels. *J. Natl Cancer Inst.*, **79**, 1261-1267.

HOLMGREN, J., LINDHOLM, L., PERSSON, B. & 8 others (1984). Detection by monoclonal antibody of carbohydrate antigen CA 50 in serum of patients with carcinoma. *Br. Med. J.*, **288**, 1479-1482.

KOPROWSKI, H., STEPLEWSKI, Z., MITCHELL, K., HERLYN, M. & FUHRER, P. (1979). Colorectal carcinoma antigens detected by hybridoma antibodies. *Somatic Cell. Genet.*, **5**, 957-972.

KOPROWSKI, H., BLASZCZYK, M., STELEWSKI, Z., BROCKHAUS, M., MAGNANI, J.L. & GINSBURG, V. (1982). Lewis blood type may affect the incidence of gastrointestinal cancer. *Lancet*, **1**, 1332-1333.

KUUSELA, P., JALANKO, H., ROBERTS, P. & 4 others (1984). Comparison of CA 19-9 and carcinoembryonic antigen (CEA) levels in the serum of patients with colorectal diseases. *Br. J. Cancer.*, **49**, 135-139.

LANGKILDE, N.C., WOLF, H., MELDGÅRD, P. & ØRNTOFT, T.F. (1991). Frequency and mechanism of Lewis antigen expression in human urinary bladder and colon carcinoma patients. *Br. J. Cancer*, **63**, 583-586.

LINDHOLM, L., HOLMGREN, J., SVENNERHOLM, L. & 5 others (1983). Monoclonal antibodies against gastrointestinal tumour-associated antigens isolated as monosialogangliosides. *Int. Arch. Allergy Appl. Immunol.*, **71**, 178-181.

MAGNANI, J.L., NILSSON, B., BROCKHAUS, M. & 6 others (1982). A monoclonal antibody defined antigen associated with gastrointestinal cancer is a ganglioside containing sialylated lacto-N-fucopentaose II. *J. Biol. Chem.*, **257**, 14365-14369.

MAGNANI, J.L., NILSSON, B., BROCKHAUS, M. & 6 others (1982). A monoclonal antibody defined antigen associated with gastrointestinal cancer is a ganglioside containing sialylated lacto-N-fucopentaose II. *J. Biol. Chem.*, **257**, 14365-14369.

MAGNANI, J.L., STEPLEWSKI, Z., KOPROWSKI, H. & GINSBURG, V. (1983). Identification of the gastrointestinal and pancreatic cancer-associated antigen detected by monoclonal antibody 19-9 in the sera of patients as a mucin. *Cancer Res.*, **43**, 5489-5492.

METZ, C.E. (1978). Basic principles of ROC analysis. *Semin. Nucl. Med.*, **8**, 283-98.

MOORE, M., JONES, D.J., SCHOFIELD, P.F. & HARNDEN, D.G. (1989). Current status of tumor markers in large bowel cancer. *World J. Surg.*, **13**, 52-59.

NILSSON, O., MANSSON, J.E., LINDHOLM, L., HOLMGREN, J. & SVENNERHOLM, L. (1985). Sialosyllactotetraosylceramide, a novel ganglioside antigen detected in human carcinomas by a monoclonal antibody. *FEBS Lett.*, **182**, 398-402.

PAGANUZZI, M., ONETTO, M., MARRONI, P. & 4 others (1988). CA 19-9 and CA 50 in benign and malignant pancreatic and biliary disease. *Cancer*, **61**, 2100-2108.

ROBERTS, P.J. (1988). Tumour markers in colerectal cancer. *Scand. J. Gastroenterol.*, **149**, suppl 50-58.

RODBARD, D. (1974). Statistical quality control and routine data processing for radioimmunoassay and immunoradiometric assays. *Clin. Chem.*, **20**, 1255-1270.

SCHOUW, Y.T., VAN DER, RUIJS, J.H.J., SEGERS, M.F.G. & 4 others (1991). Initial assessment of diagnostic markers, illustrated by CEA for colorectal carcinoma. Submitted.

SCHWARTZ, M.K. (1990). Advances in the use of tumor markers. *Compr. Ther.*, **16**, 51-7.

STAAB, H.J., BRÜMMENDORF, T., HORNUNG, A., ANDERER, F.A. & KIENIGER, G. (1985). The clinical validity of circulating tumor-associated antigens CEA and CA 19-9 in primary diagnosis and follow-up of patients with gastrointestinal malignancies. *Klin. Wochenschr.*, **63**, 106-115.

SWETS, J.A. (1973). The relative operating characteristic in psychology. *Science*, **182**, 990-1000.

SUNDARAM, S.G., MANIMEKALAI, S., UNNI, S. & 4 others (1987). Evaluation of antigen CA-195 for the detection of colon cancer. *J. Tumor Marker Oncol.*, **2**, 315-318.

WATKINS, W.M. (1980). Biochemistry and genetics of the ABO, Lewis, and P blood group systems. In Harris, H. & Hirschhorn, K. *Advances in Human Genetics*. Plenum Press: New York.

WEINSTEIN, M.C. & FEINBERG, H.V. (1980). *Clinical Decision Analysis*. W.B. Saunders Company: Philadelphia.

WOBBES, TH., THOMAS, C.M.G., SEGERS, M.F.G. & NAGENGAST, F.M. (1992). Evaluation of seven tumour markers (CA-50, CA-19-9, CA-19-9 TruQuant, CA-72.4, CA-195, CEA and TPA) in pretreatment sera of patients with gastric ulcer. *Cancer*, **69**, 2036-2041.